# Ultrastructural Study of Alveolar Epithelial Type II Cells by High-Frequency Oscillatory Ventilation

**DOI:** 10.1155/2013/240659

**Published:** 2013-12-10

**Authors:** Xiaofei Qin, Wanhai Fu, Youwei Zhao, Qiong Meng, Chuming You, Qiuming Yu

**Affiliations:** Department of Pediatrics, Guangdong Second Provincial People's Hospital, No. 1 Shiliugang Road, Haizhu District, Guangzhou 510317, China

## Abstract

Alveolar epithelial type II cells (AECIIs) containing lamellar bodies (LBs) are alveolar epithelial stem cells that have important functions in the repair of lung structure and function after lung injury. The ultrastructural changes in AECIIs after high-frequency oscillatory ventilation (HFOV) with a high lung volume strategy or conventional ventilation were evaluated in a newborn piglet model with acute lung injury (ALI). After ALI with saline lavage, newborn piglets were randomly assigned into five study groups (three piglets in each group), namely, control (no mechanical ventilation), conventional ventilation for 24 h, conventional ventilation for 48 h, HFOV for 24 h, and HFOV for 48 h. The lower tissues of the right lung were obtained to observe the AECII ultrastructure. AECIIs with reduced numbers of microvilli, decreased LBs electron density, and vacuole-like LBs deformity were commonly observed in all five groups. Compared with conventional ventilation groups, the decrease in numbers of microvilli and LBs electron density, as well as LBs with vacuole-like appearance and polymorphic deformity, was less severe in HFOV with high lung volume strategy groups. AECIIs were injured during mechanical ventilation. HFOV with a high lung volume strategy resulted in less AECII damage than conventional ventilation.

## 1. Introduction

The use of mechanical ventilation in clinical practice has increased the survival rate of newborns. However, respiratory and neurological complications or sequelae have increased because of ventilator-induced lung injury (VILI), a phenomenon caused or aggravated by mechanical ventilation. Alveolar epithelial type II cells (AECIIs) are alveolar epithelial stem cells targeted in acute lung injury (ALI) and acute respiratory distress syndrome (ARDS) or VILI. The structural and functional changes in AECIIs are related to ALI, ARDS, and VILI [1, 2]. The normal function of AECIIs relies on normal cytomorphology.

High-frequency oscillatory ventilation (HFOV) is a lung protective ventilation strategy in newborns with ALI or ARDS. However, data on the effect of HFOV on the ultrastructural features of AECIIs are limited. The practical application value of HFOV is still controversial. This study used a newborn piglet model with ALI to study the effect of conventional ventilation and HFOV with a high lung volume strategy on AECIIs. The ultrastructural features of AECIIs after HFOV with a high lung volume strategy in a newborn piglet model with ALI were evaluated.

## 2. Materials and Methods

### 2.1. Animals and Surgery

Fifteen newborn piglets (≤3 days old, weighing 1.0 kg to 1.97 kg) were used. All animal experiments were performed with the approval of the Guangdong Second Provincial People's Hospital Animal Care and Use Committee (2009-XEK-028).

Piglets were given 10% chloral hydrate (1 mL/kg) orally and placed in a supine position under an infant radiant warmer. The body temperature of the animals was maintained in the range of 38.0°C to 39.5°C. Catheters were inserted in the axillary vein and the femoral artery for injecting medication and fluids, as well as for blood gas analysis and arterial blood pressure monitoring. The maintenance fluids included continuous infusion of 0.9% saline solution containing 5% dextrose (120 mL/kg/d). A dopamine infusion (5 *μ*g/kg/min) was continuously administered. The piglets were anesthetized using diazepam (0.5 mg/kg, intramuscularly) and ethyl carbamate (0.6 g/kg, intravenously). The piglets were intubated with endotracheal tubes (4.0 mm internal diameter) and then ventilated using conventional ventilation (Servo-i; Maquet, Solna, Sweden) or HFOV. Each piglet was intravenously infused with a bolus of cefotiam hydrochloride (100 mg/kg).

Using the pressure control mode, we set the initial ventilator settings to a positive end-expiratory pressure (PEEP) of 2 cm H_2_O, a peak inspiratory pressure of 10 cm H_2_O, an inspiratory-to-expiratory ratio of 1 : 2, and a fraction of inspired oxygen (FiO_2_) of 0.30. The breathing frequency was set to 25 breaths/min to 30 breaths/min and then adjusted to maintain the pressure of arterial carbon dioxide (PaCO_2_) in the normal range (35 mm Hg to 45 mm Hg).

### 2.2. ALI and Treatment Regimens

ALI was induced by lavaging the whole lung with normal saline. During lavage, all piglets were ventilated by a conventional mechanical ventilator using the pressure control mode. Warmed (37°C) normal saline (35 mL/kg) was instilled into the lung via the endotracheal tube. Saline was allowed to remain in the lung for 10 s before removal. Lung lavage was repeated at 5 min intervals until the pressure of arterial oxygen was below 100 mm Hg for 60 min at the following ventilator settings: peak inspiratory pressure, 24 cm H_2_O; PEEP, 6 cm H_2_O; inspiratory-to-expiratory ratio, 1 : 2; FIO_2_, 1.0; and breathing frequency, 35 breaths/min.

After lung injury was established, the piglets were randomly assigned into five study groups (with three piglets in each group): control (receiving no ventilation), conventional ventilation for 24 h, conventional ventilation for 48 h, HFOV for 24 h, and HFOV for 48 h. The control piglets were sacrificed by overdosing with 10% potassium chloride under deep anesthesia. In the conventional ventilation group, the piglets were ventilated using a conventional mechanical ventilator (Servo-i) in the pressure control mode at the following ventilator settings: peak inspiratory pressure, 20 cm H_2_O; PEEP, 4 cm H_2_O; inspiratory-to-expiratory ratio, 1 : 2; and FIO_2_, 1.0. The breathing frequency was set to 25 breaths/min to 30 breaths/min and adjusted to maintain PaCO_2_ in the normal range (35 mm Hg to 45 mm Hg). In the HFOV group, the piglets were placed in HFOV (SLE-5000; Tokibo, Tokyo, Japan) with an oscillatory frequency of 10 Hz, a fractional inspiratory time of 33%, and an FIO_2_ of 1.0. The mean airway pressure was set to 2 cm H_2_O higher than that in conventional ventilation, which indicated a high lung volume strategy in HFOV. The amplitude was set to the range of 20 cm H_2_O to 25 cm H_2_O and adjusted to maintain PaCO_2_ in the normal range (35 mm Hg to 45 mm Hg). During ventilation, FIO_2_ was decreased by 10% every 6 h up to 40%. At the end of ventilation, the piglets were sacrificed by overdosing with 10% potassium chloride under deep anesthesia. The lungs were immediately removed, and samples (1 mm^3^) were obtained from the lower parts of the right lung and fixed with 2.5% glutaraldehyde.

### 2.3. Transmission Electron Microscope

The samples were prefixed in 2.5% glutaraldehyde, postfixed in 1% osmic acid, and then embedded in Epon 812. Ultrathin sections prepared using an ultramicrotome were stained with uranyl and lead citrate. AECIIs were examined under a transmission electron microscope by two blinded, independent observers.

## 3. Results

### 3.1. Ultrastructural Changes in AECIIs from the Control Group

**Figure 1:**
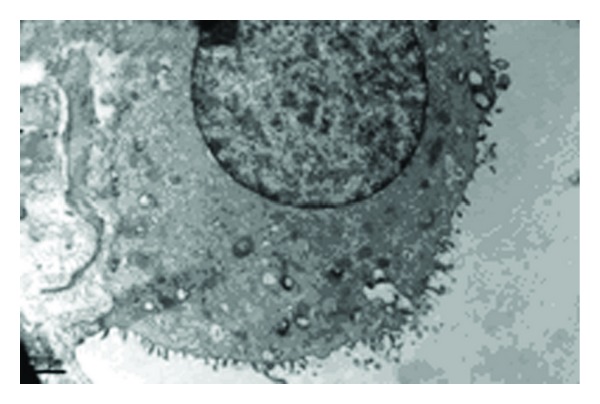
Ultrastructural changes of AECIIs in control group (×9700). The nuclei were round and clear. Some LBs were presented as decreased in electron density and vacuole-like deformity; some LBs with density uniformity and ring-like arrangement were shown.

The ultrastructure of AECIIs showed tight interaction with the basal membrane and AECIs. The nuclei were round and clear, and the chromatin inside each nucleus was homogeneous. Lamellar bodies (LBs) with uniform density and ring-like arrangement were present. Some LBs showed decreased electron density and vacuole-like deformity. Microvilli were displayed distinctly (Figure 1).

### 3.2. Ultrastructural Changes in AECIIs from the Conventional Ventilation Group

In both the conventional ventilation 24 h and 48 h groups, LBs were arranged around the nucleus in reduced numbers and with decreased electron density. Some AECIIs showed shrunken nuclei containing nonhomogenous, condensed chromatin.

**Figure 2:**
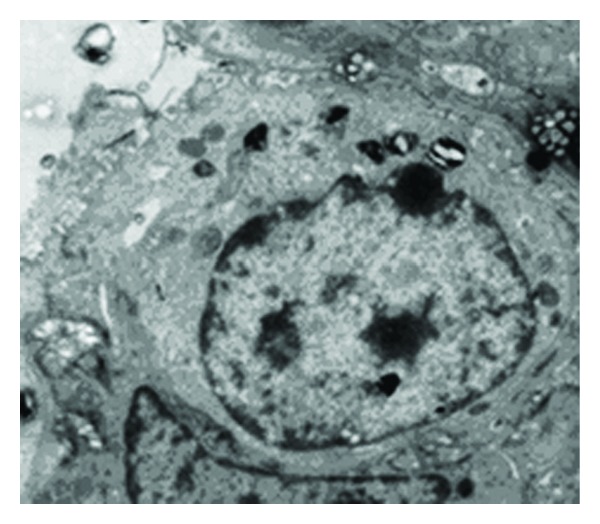
Ultrastructural changes of AECIIs in conventional ventilation 24 h group (×5800). The secretion of AECII discharged into alveolar space was observed.

In the conventional ventilation 24 h group (Figure 2), AECIIs interacted with the basal membrane and loose AECIs. AECII secretions were discharged into the alveolar space, and naive AECIIs were observed.

**Figure 3:**
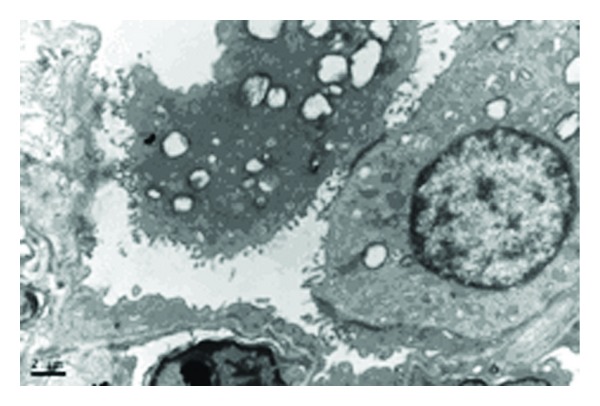
Ultrastructural changes of AECIIs in conventional ventilation 48 h group (×9700). AECII without nucleus was observed. Microvilli were irregularly arranged.

At 48 h, some AECIIs were dislodged from the basal membrane (Figure 3). Some AECIIs did not have nuclei and showed LB with vacuole-like and polymorphic deformity. Giant LB and irregularly arranged microvilli were the major manifestations.

### 3.3. Ultrastructural Changes in AECIIs from the HFOV Group

**Figure 4:**
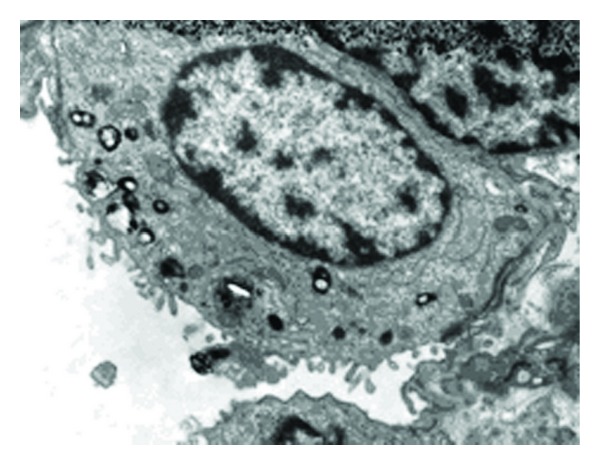
Ultrastructural changes of AECII in HFOV 24 h group (×9700). The juxtaposition of AECII to basal membrane and AECI was close.

In the HFOV 24 h group, the juxtaposition of AECIIs to the basal membrane and AECIs was close (Figure 4). The changes in LB (vacuole-like appearance and polymorphic deformity) were less severe than those in the conventional 24 h group. The nuclei and karyosome were round and clear. The chromatin inside the nuclei was concentrated and condensed.

**Figure 5:**
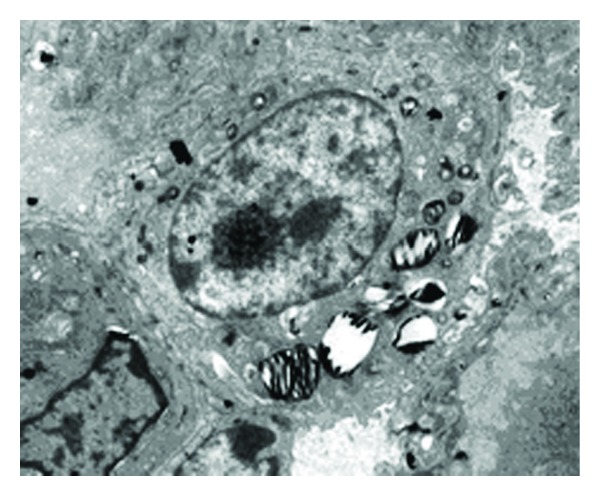
Ultrastructural changes of AECIIs in HFOV 48 h group (×5800). The incompletely vacuole-like deformity in LB was presented.

Except for small numbers of AECIIs, most of the cells interacted tightly with the basal membrane and AECI under continuous ventilation on HFOV up to 48 h (Figure 5). The nuclei were round and regular. Incomplete vacuole-like deformity in LB was also observed.

## 4. Discussion

AECIIs cover approximately 4% of the mammalian alveolar surface but constitute 15% of all lung cells as multifunctional cells. AECIIs participate in the defense and pathogenesis of infection. AECIIs also perform various important functions within the lungs, including regulation of surfactant metabolism, ion transport, and alveolar repair in response to injury. Clinically, obtaining lung tissue from neonates on a mechanical ventilator to study AECIIs is difficult. Therefore, we used newborn piglets as models to observe the ultrastructural features of AECIIs in animals with lung injury treated by conventional ventilation or HFOV.

Figure 3 shows that AECIIs display the following structural features typical of cell damage with increasing ventilation time in ventilated lungs: nuclei shrunk in size or without a nucleus, karyopyknotic in shape, cell dissociated and dislodged from the basal membrane and AECIs, LBs decreased in electron density and numbers, and LBs with vacuole-like and polymorphic deformity. As illustrated in Figure 2, AECII secretion was discharged into the alveolar space. Figures 2 and 3 show the different degrees of damage in AECIIs (apoptosis, necrosis, and degeneration) during mechanical ventilation, which may lead to insufficiency of pulmonary surfactant synthesis and secretion. The reasons for these observations are discussed. On the one hand, stretched AECIIs and alveolar macrophages promote the release of proinflammatory mediators in mechanical ventilation [3, 4]. On the other hand, the structure and function of AECIIs are damaged after lung injury, and pulmonary surfactant synthesis and secretion are decreased [5–8], which downregulate host immunologic defense and decrease host anti-inflammatory ability. This result agrees with our previous work. AECII damage is aggravated as observed under transmission electron microscope, and pulmonary inflammation is deteriorated [9]. As the most important part of the innate immune system of the lung, AECIIs boost the activity of phagocytes [10–12]. Alveolar macrophages phagocytose apoptotic polymorphonuclear neutrophils, which not only depress the secretion of proinflammatory cytokines, such as interleukin-1*β*, interleukin-8, interleukin-10, and tumor-necrosis-factor-*α*, but also promote the secretion of anti-inflammatory mediators, such as transforming growth factor *β*1 [13, 14]. Alveolar macrophages and other phagocytes eliminate apoptotic cells by recognizing molecules, such as CD44, on apoptotic cell membrane surface [15, 16]. These molecules are important in the inflammatory response development of ALI/ARDS and VILI. ALI/ARDS and VILI are aggravated, when the elimination of apoptotic polymorphonuclear neutrophils is reduced or slowed down. AECIIs improve the activity of alveolar macrophages by synthesizing and secreting pulmonary surfactants [17]. AECIIs increase the activity of phagocytes by pulmonary surfactants [17]. The metabolism of pulmonary surfactants is related to synthesis and secretion by AECIIs. Macrophages, such as alveolar macrophages and polymorphonuclear neutrophils, also participate in pulmonary surfactant clearance [18, 19]. In the presence of ALI/ARDS and VILI, alveolar macrophages, polymorphonuclear neutrophils, and inflammatory cells are collected in the lung to decrease and inactivate lipids in pulmonary surfactants [20–22]. The susceptibility of lung tissue to inflammation increases [23]. These factors may aggravate or cause an imbalance between inflammatory response and anti-inflammatory response. They also attract a large number of proinflammatory factors and mediators that accumulate in lung tissue. Lung injury is aggravated by inflammatory cascade amplification reaction and further damages AECIIs.

The results of transmission electron microscopy showed that the juxtaposition of AECIIs in the HFOV 48 h group to the basal membrane and AECIs was closer in ventilated lungs and with the extension of ventilation time. In addition, the electron density of LB in the HFOV 48 h group decreased less than that in the conventional ventilation 48 h group. Lamellar structure was clearly observed in the HFOV 48 h group. This result suggested that HFOV caused less damage on AECIIs than conventional ventilation. Stüber et al. [24] found that high ventilation pressure can increase proinflammatory mediators, such as interleukin-6, compared with low ventilation pressure. Lung protective ventilation strategy reduces proinflammatory mediators [25–27] and surfactant protein D [28, 29]. HFOV is regarded to have protective effects on the lungs because of its small tidal volumes, low peak inspiratory pressure, high PEEP, and fixed mean airway pressure. HFOV results in a low number of proinflammatory mediators in clinical [30] or animal [31, 32] trials. HFOV reduces AECII injury by relieving inflammation. Moreover, under HFOV, uniformly dilated alveoli reduce AECII damage by force resulting from repeated opening and closing of alveoli. Our previous study [9] showed that HFOV reduces polymorphonuclear neutrophil infiltration, hemorrhage, alveolar edema, and hyaline membrane formation with improved oxygenation. HFOV participated in maintaining the structural integrity and functional stability of AECIIs and lung tissue.

AECIIs were injured when newborn piglets were ventilated. AECIIs from piglets under HFOV with a high lung volume strategy showed better structural integrity compared with AECIIs from piglets maintained under conventional ventilation.

## 5. Limitations

The samples of both the control and study groups were small in this study. We concentrated only on the ultrastructural changes in AECIIs following HFOV with a high lung volume strategy or conventional ventilation in a newborn piglet model with ALI. However, our results were all descriptive analysis and lacked quantitative presentation of data. Notwithstanding its limitations, this study is interesting and suggests that AECIIs are injured during mechanical ventilation and that HFOV with a high lung volume strategy results in less AECII damage than conventional ventilation. The sample size and quantitative analysis must be, respectively, increased and broadened to study the possible mechanisms of HFOV to minimize injury.
